# Lifeworlds in pain: a principled method for investigation and intervention

**DOI:** 10.1093/nc/niad021

**Published:** 2023-09-13

**Authors:** Abby Tabor, Axel Constant

**Affiliations:** Faculty of Health and Applied Sciences, University of the West of England, Frenchay Campus, Coldharbour Ln, Stoke Gifford, Bristol BS16 1QY, UK; Centre for Pain Research, University of Bath, Claverton Down, Bath BA2 7AY, UK; Department of Engineering and Informatics, The University of Sussex, Chichester 1 Room 002, Falmer, Brighton BN1 9QJ, UK

**Keywords:** theories and models, psyciatry, embodiment, pain, anthropology, bayesian inference

## Abstract

The experience of pain spans biological, psychological and sociocultural realms, both basic and complex, it is by turns necessary and devastating. Despite an extensive knowledge of the constituents of pain, the ability to translate this into effective intervention remains limited. It is suggested that current, multiscale, medical approaches, largely informed by the biopsychosocial (BPS) model, attempt to integrate knowledge but are undermined by an epistemological obligation, one that necessitates a prior isolation of the constituent parts. To overcome this impasse, we propose that an anthropological stance needs to be taken, underpinned by a Bayesian apparatus situated in computational psychiatry. Here, pain is presented within the context of lifeworlds, where attention is shifted away from the constituents of experience (e.g. nociception, reward processing and fear-avoidance), towards the dynamic affiliation that occurs between these processes over time. We argue that one can derive a principled method of investigation and intervention for pain from modelling approaches in computational psychiatry. We suggest that these modelling methods provide the necessary apparatus to navigate multiscale ontology and epistemology of pain. Finally, a unified approach to the experience of pain is presented, where the relational, inter-subjective phenomenology of pain is brought into contact with a principled method of translation; in so doing, revealing the conditions and possibilities of lifeworlds in pain.

## Introduction

On the surface, the experience of pain appears paradoxical (Bradley 2021). It is ubiquitous, yet idiosyncratic, both basic and complex; by turns necessary and devastating. Pain is poised tantalisingly across body and mind, determined by context, yet defined by the individual. The consequences of this can be observed from the metaphorical to the practical: pain resists definition, classification and, crucially, effective treatment when it persists (Williams et al. 2020). Our understanding of pain, from a scientific perspective, has broadened considerably in the last 10 years (Moayedi and Davis 2013; Williams 2016; Karos et al. 2018; Seymour 2019; Stilwell and Harman 2019; Tabor and Burr 2019; Kiverstein et al. 2022). No longer constrained to the detection of damage, the experience of pain spans biological, psychological and sociocultural realms (Raja et al. 2020). With increasing precision, the parameters that influence our experience of pain have been identified across multiple disciplines (Wiech 2016; Buchbinder et al. 2018; Johnston et al. 2019). Yet, even with the scope that different expertise has afforded, the effective integration of these parameters remains elusive (Flor and Turk 2015).

The biopsychosocial (BPS) model (Engel 1977) stands as the principal frame in which diffuse disciplinary knowledge is drawn together in the domain of health; a pervasive philosophy that continues to influence the way researchers and clinicians alike investigate and intervene (Borrell-Carrió et al. 2004). With its roots in systems theory, the premise of the BPS was to reject biomedical reductionism in favour of a multidimensional, integrative approach to health and illness. It is argued, however, that the BPS has failed to realise these conceptual underpinnings (Benning 2015), in both theory (Pilgrim 2002; Ghaemi 2009) and practice (Cohen 1993; Suls and Rothman 2004; Kecmanovic and Hadzi-Pavlovic 2010). Pertinently, a neglect is observed in the way in which the BPS is able to accommodate the influence of large sociocultural units on the one hand, and the subjective reality of patients on the other (Benning 2015). Its inability to function across multiple scales and between multiple levels of influence—a characteristic demanded by complex conditions—is a fundamental limitation of the model. For Engel,

‘[e]ach system as well implies qualities and relationships distinctive for that level of organization and requires unique criteria for study and explanation. In no way can the methods and rules appropriate for the study and understanding of the cell as cell be applied to the study of the person as person or the family as family. Similarly, the methods needed to identify and characterize the components of the cell have to be different from those required to establish what makes for the wholeness of the cell’ (Engel 1981, 106).

The BPS model imposes that prior to an integrated consideration of the whole system, there must be isolation. And although such systems ought to be understood as nested across their scales of organisation, according to this approach they cannot be explained with a single integrative strategy. Put another way, under the BPS model, only the ontology of a disorder cuts across levels, not its epistemology; ontology being concerned with ‘what things are’, and epistemology being concerned with ‘how we come to know’ that which is (Bateson 1971, 442). Moreover, in pain research, although intended as a philosophy of care, the translation of the BPS model in theory and practice plays out under the shadow of pain’s paradoxes (Stilwell and Harman 2019). That is, where integration is sought, compartmentalised targets are found; without a truly integrative model to guide action (Borrell-Carrió et al. 2004), bio-psycho-social elements are pursued with ‘eclectic freedom’ (Ghaemi 2009), reduced to disembodied risk factors to be separated, investigated and treated (Moseley and Flor 2012; van Hecke et al. 2013; Johnston et al. 2019; Apkarian 2011). The consequence of an inadequate integrative multiscale and multilevel approach to healthcare generally and pain specifically is that despite great advances in our knowledge of pain, effective treatment remains lost in translation. We are faced with therapies that are not efficacious (Williams et al. 2020; O’Connell et al. 2023), a global opioid crisis (Marshall et al. 2019), and an increasing prevalence of persistent pain conditions that constitutes an epidemic (Gatchel 2015; Domenichiello and Ramsden 2019).

In response, we first invite a return to an anthropological perspective on the experience of pain (Good et al. 1994): centred on individual experience but inseparable from the broader socio-cultural frame. Second, we provide a structural model that guides multiscale action: a computational approach grounded in Bayesian inference. Accordingly, the goal of this paper is to outline a principled method of investigation and intervention for the experience of pain, embedded and embodied in a lifeworld.

This paper proceeds by centring on the phenomenological character of pain, while acknowledging that the experience can be described across multiple scales of organisation (i.e. it is a multiscale phenomenon). And for this reason, requires explanations that can be pitched across several levels (i.e. multilevel explanations). In the following section of this paper, we set out the necessary grounding developed through an anthropological stance to pain, understood as a relational experience. We propose that the experience of pain emerges through on-going ‘correspondence’ within a particular lifeworld (Ingold 2017). This allows us to describe the microcosm of pain experience—the local lifeworld of the individual, in relation to the macrocosm of the sociocultural niche, not isolated but subsumed within one another. Next, we present a framework borrowed from computational psychiatry to offer an apparatus that captures the multiscale nature of afflictions configured at the level of subjective experience (i.e. mental disorders (Kirmayer and Young 1999)) and an explanatory framework for a multilevel reading of such afflictions. We then propose ‘A unified approach’: detailing an integrated translation, directed towards investigation and intervention within the field of persistent pain; its proposed efficacy illustrated with a case study, drawn from anthropological inquiry, and translated through a computational psychiatry approach. It is suggested that existential anthropology and computational psychiatry together promote an embodied and embedded approach for a unified conceptualisation of pain; the two approaches underpinned by a commitment to investigate the conditions and possibilities of systems in the world.

## An anthropological stance

The experience of pain is well suited to anthropological inquiry, with detailed ethnographies providing valuable insight into the human condition (Good et al. 1994; Kleinman 1997; Honkasalo 2001). Such accounts reveal the sociocultural shaping of pain, an ecosocial phenomenon, i.e. fundamentally relational in its makeup (Kirmayer 2008; Karos et al. 2018). Yet, these contributions have been largely neglected in contemporary pain inquiry, which has favoured objective accounts, informed by cognitive-behavioural (Eccleston and Crombez 1999; Vlaeyen et al. 2016), neuroscientific (Wager et al. 2013; Davis 2019) and epidemiological pursuits (Johnston et al. 2019), increasingly in the service of precision medicine (Nijs et al. 2021). In taking an anthropological turn, we assert that the success of such approaches will be determined by the attention paid to the embedded individual (Ziegelstein 2017).

As such, we define an anthropological stance in terms of an active endeavour, a mode of inquiry that approaches the experience of pain as part of an individual’s local lifeworld. In keeping with 4E approaches to cognition (Varela et al. 2017; Colombetti 2017; Gallagher 2023), the experience of pain is investigated as embodied, enacted, embedded and extended in a dynamic sociocultural niche (Kleinman 1992; Csordas 2002; Jackson 2012; Tabor et al. 2017). Additionally, this position draws on a rich history of existential approaches to suffering (Kleinman 1997; Jackson 2019) and brings them into contact with a detailed phenomenological approach to experience (Merleau-Ponty 1945; Dreyfus 1990; Jackson 2005). Under this gaze, pain cannot be separated from the individual nor a dynamism of being-in-the-world (Merleau-Ponty 1945; Dreyfus 1990).

A crucial extension gained by adopting an anthropological stance, beyond phenomenological and embodied approaches, is the conceptual reach; capturing the way in which pain and suffering has changed over time, across generations, cultures and social networks, as well as across a single lifespan. It provides the means to situate the individual in the present, informed by the past, and oriented towards an uncertain future. We now explore these means, through the conceptual metaphors of ‘lifeworlds’, ‘lines’ and ‘correspondence’.

### Lifeworlds and lines

Our starting point is one of phenomenological grounding (Husserl 1970), from which our experiences are brought forth through an ongoing engagement with the world (Merleau-Ponty 1945). In following the existential anthropology of Jackson (2005), we extend this phenomenological base to incorporate a detailed reckoning of a dynamic ecosocial world; in short, a lifeworld (Jackson 2012). Our lifeworlds are not settled, but comprise a landscape through which we are able to continually negotiate a sense of ourselves, always in relation to others (Jackson 2005, 2012). A lifeworld presents an embodied history in transition, situated in the present but oriented towards the possibilities of the future; it is ‘a field charged with vitality and animated by struggle’ (Jackson 2012, 7). A lifeworld can be considered an evolving entanglement of lines, which reach out, beyond single systems, forming a meshwork. In evoking the meshwork, as Ingold does (Ingold 2015), we depart from the dominant metaphor of network (Latour 2005). Where the network makes distinct the components of a system and its lines of connection, a meshwork transfigures a system into ‘lines of becoming’ (Deleuze and Félix 1987), not curtailed by set points of beginning or end, the lines themselves become the focus, differentially corresponding with each other as they ‘bod(y) forth’ (Lefebvre 1974; Ingold 2011; 2016, 2017). These lines are indicative of system processes, from the microscopic to the macroscopic (e.g. immune dynamics, psychological states, family responsibilities and workplace identity).

In other words, the lifeworld sets the bounds on what is afforded, ‘for good and for ill’, to a particular system at a given time (Gibson 1979), and the particular entanglement of lines determines the shape of that lifeworld. It is in this formulation of lifeworlds that allows the negotiation of new possibilities within the ‘subjective in-between’ (Arendt 1958), i.e. entanglements of lines that are brought under tension or loosened through an ongoing process of correspondence.

### Correspondence

Correspondence, as described by Ingold (Ingold 2015) refers to the affiliations between the lines of a lifeworld. Correspondence extends to incorporate the relation between lifeworlds, reaching out across a meshwork (Ingold 2017); affiliations that wax and wane as living systems intertwine with each other and their environment over time. It demands a focus of attention on what it is to ‘be-in-the-world’ (Heidegger 1962): in tension with other organisms. In this way, correspondence denotes the way in which the processes of a lifeworld influence each other.

### Correspondence and lifeworlds

Correspondence determines the texture of our lifeworlds. An illustrative example can be observed during jazz improvisation. When a saxophonist plays, there is, necessarily, an interaction between the musician and their instrument, yet this point of connection is not correspondence. Rather, the melody that emerges from this coming together is what is meant by correspondence (Ingold 2011). Furthermore, as the bassist, trumpeter, trombonist and clarinettist join in, each with their own melody in response to the saxophonist’s ‘question’, a polyphonic experience results. Polyphony, emerging through improvised jazz can be thought of as a particular lifeworld composed of multiple lines in correspondence. As the dynamic between different musicians changes over time, with melodies rising to prominence and then receding, the correspondence is altered; an evolving process that shapes and reshapes the shared musical lifeworld. Here, each melody is a line in a polyphonic lifeworld, and it is through the correspondence of these lines that the lifeworld derives its texture. It necessitates agile attending, deployed in sympathy with one another; negligence risks discordance, incoherence, and in a musical context, noise.

### Lifeworlds and correspondence in pain

In applying this conceptualisation to pain, the lifeworld must capture the multiscale aspects of the individual (micro, meso and macro), while extending beyond the individual to encompass the broader sociocultural milieu (past, present and future). Here, the lifeworld of an individual in pain, whether acute or chronic, is considered an entanglement of lines that reach beyond bodily bounds, affiliated in a way that reflects dynamic correspondence. Manifesting in a particular phenomenology for the individual, the lines of the lifeworld reach across evolutionary and life histories, biological traces, psychological repertoires, social interactions, cultural expectations, political agendas and global constraints. These lines are not separate but interwoven to form a meshwork reflective of a supersystem, in which the lifeworld for the individual in pain, is embroidered.

In most cases, the experience of pain is short-lasting, a lifeworld reshaped through the changing correspondence of its lines. Yet, for a significant number of people, the experience of pain persists (Fayaz et al. 2016). In the present context, and in keeping with recent embodied (Tabor et al. 2017; Stilwell and Harman 2019) and predictive accounts of pain (Büchel et al. 2014; Tabor and Burr 2019; Kiverstein et al. 2022), this apparent ‘stickiness’ of aversive experience (Borsook et al. 2018) is related to a continued anticipation of threat to the self. The persistent anticipation of threat involved in pain has been attributed to various mechanisms, from maladaptive learning and aberrant precision allocation in reward processing (Seymour 2019), to an altered landscape of affordances (Stilwell and Harman 2019). In common, these accounts describe the complex componentry of anticipatory protective action, from neural networks to environmental contingencies. In taking an anthropological stance, however, our attention is trained on the ways in which these processes, or lines of a lifeworld, correspond for the individual in pain over time. The greater the affiliation between lines (e.g. a physiological stress response, historical trauma, completing assignments and fulfilling socio-cultural expectations), the greater influence this entanglement of lines has on the shape of the lifeworld. It pulls tight, narrowing the lifeworld in a certain direction—a tangled skein. As a consequence, the landscape is altered, what is afforded to the individual is narrowed to reflect ‘what is at stake’ for them at that time (Miller et al. 2020). At risk for the individual in pain when correspondence constricts, is incoherence: a discordance between their local lifeworld (narrowed to reflect a need to protect), and the wider niche, soliciting engagement.

In taking this perspective, the traditional boundaries between adaptive and maladaptive pain become less distinct. Here, the experience of pain *always* reflects multiscale processes in correspondence at any given time, within a particular lifeworld. These processes span proximal (developmental, e.g. endocrine function) and ultimate (evolutionary, e.g. threat anticipation) timescales (Constant et al. 2022), influencing the current experience of pain. In this sense, detailing the processes of a lifeworld should determine investigation and intervention, irrespective of the *duration* of pain. However, the ability to contextualise the lifeworld, which considers proximal and ultimate forces in correspondence, is fraught with difficulty. Previous attempts at navigating this translation have thus far proved either overwhelmingly complex (Fabrega and Tyma 1976) or limited by simplicity (e.g. as with the BPS). A further step is required to overcome the translation gap. We have proposed that an anthropological stance provides an impetus for action, yet an apparatus is needed to enable action to be taken. To address this, we turn, in the next section, to the domain of computational psychiatry, where advances in the application of predictive modelling (Kirmayer 2019; Gómez-Carrillo and Kirmayer 2023) provide one such apparatus.

## Multiscale ontology and multilevel epistemology in computational psychiatry

In the introduction, we suggested that instrumental approaches to health, specifically the BPS model, do not provide an integrative account of pain. Instead, it is implemented in a way that isolates constituent parts, across scales of influence, in an attempt to understand the whole. This is a problem for the view advocated in this paper, which seeks to provide a principled approach to pain understood as a unified phenomenon. We have suggested that an anthropological stance provides a systems approach that does not reduce pain to its componentry but describes the processes through which it may emerge and evolve. To complement this prospective approach, we now provide the apparatus for translation, appealing to the underlying epistemology and ontology of computational psychiatry.

Computational psychiatry (Corlett and Fletcher 2014; Friston et al. 2014) is a multidisciplinary domain of research in theoretical neuroscience that analyses clinical and behavioural data to design computer models of the environmental, social and neurobiological causal networks underpinning mental disorders (Huys et al. 2016; Gauld et al. 2021). Models in computational psychiatry are used to analyse, simulate and forecast the way behavioural and psychological symptoms are generated, thereby providing promising methods for phenotyping and nosology (Schwartenbeck and Friston 2016) as well as to appraise prognosis (Constant et al. 2021). As a research program, computational psychiatry contains three subdomains (Gauld et al. 2021): (i) digital psychiatry—the creation of digital interfaces to gather more ecologically valid types of data, such as ecological momentary assessment data (Shiffman et al. 2008); (ii) big data psychiatry—the use of machine learning and artificial intelligence to treat large clinical data sets; and (iii) modelling psychiatry—the use of reinforcement learning, dynamical system theory and Bayesian methods to provide biobehaviorally plausible models of symptoms of mental disorders. Empirically, Bayesian modelling methods of computational psychiatry have been used for biophysical modelling of neuronal processes (Isomura 2022; Kagan et al. 2022), behavioural and social interactional modelling of decision making under uncertainty—to find computational markers of mental disorders (Cullen et al. 2018; Smith et al. 2020a, 2020b, 2020c, 2021c; Constant et al. 2021), and for psychosocial (i.e. social, psychological and cultural) modelling—the modelling of linguistically held beliefs and expectations—to predict treatment adherence and appraisal (Smith et al. 2021a).

Biophysical, Behavioural and Psychosocial models reflect the multi-scale ontology of Bayesian modelling psychiatry; psychiatric phenomena such as mental disorders being configured at the sociocultural level, the psychological or phenomenological level, and at the biophysical level (Table 1). Bayesian modelling psychiatry also provides an epistemology—a theory of ‘how we come to know’ psychiatric phenomena, which is in turn multilevel. One way to present the epistemology of Bayesian modelling psychiatry is by appealing to David Marr’s levels of analysis (Marr 2010; Corlett and Fletcher 2014).

**Table 1.** Epistemology and ontology of psychiatric phenomena

		Multi-scale ontology		
		Biophysical scale model	Behavioural and social interactional scale model	Psychosocial scale model
Multi-level epistemology	Computational level	Inference problem		
	Algorithmic level	Bayesian inference		
	Implementation level	E.g. (neuro)physiology	E.g. behavioural patterns/posture	E.g. language/healthcare systems

Psychiatric phenomena, from the computational point of view, ought to be studied as a failure to solve the fundamental problem that systems like us must solve—that of inferring the causes of sensations, with Bayesian inference being the computational specification of the problem of inferring the causes of sensations. At the algorithmic level—the level at which one decides how to solve the computational problem, this problem is solved with Bayesian inference. And at the implementation level, Bayesian inference would be realised by the dynamics of the system of interest (e.g. the (neuro)physiology of a system configured at the biophysical level). Importantly, Bayesian inference at the algorithmic level can be used to interpret the working of many different mechanisms whose dynamics at the implementation level would conform to Bayesian inference. Under the present framework, for the sake of simplicity, only the Bayesian algorithm is reflected at the algorithmic level. However, a complete approach based on computational psychiatry could consider a variety of algorithms (e.g. machine learning, natural language processing, dynamical systems, etc.).

Through a unified ‘Bayesian’ ontology and epistemology, computational psychiatry can, in principle, provide the apparatus (e.g. hierarchical models) (Friston et al. 2017a; Ramstead et al. 2018; Badcock et al. 2019), that can facilitate the coherent translation of the dynamic correspondence between system processes for investigation and intervention. That is, a weighted appraisal of correspondence across scales and levels that guides relevant action. Note that Marr’s levels of analysis function as a general guide to the inquiry on the computational functioning of an entity configured at any ontological scale. For instance, when considering the hierarchical approach of the BPS model, one could apply Marr’s strategy for each of the levels (e.g. analyse the person out of the biosphere as an entity that can be described with multiple levels of analysis). Marr’s way of approaching levels of analysis is thus consistent with the BPS way of isolating components of the hierarchy to treat them as incommensurable. As such, we do not claim that using Marr’s levels of analysis overcomes that limitation of the BPS. Instead, by evoking the BPS model through a Marrian analysis, we are able to appeal to Bayesian modelling methods that transcend hierarchical levels. These methods allow for the comparison of activity—optimal and suboptimal—at each level by modelling them as a set of parameters (e.g. likelihood, prior, and prior preferences in the case of some models (Friston et al. 2017b) whose posterior probability can be assessed with scale independent measures (e.g. model evidence). In other words, one can apply a Marrian epistemological analysis ‘within’ each level, and each ontological level can be modelled using a Bayesian modelling approach. This step makes each level commensurable, thereby revealing a unified structure through which processes correspond for targeted investigation and intervention.

It is important to note that in recent years, computational approaches in the field of pain science have gained traction. Applied to both brain-based mechanisms and behavioural data, these accounts have identified the predictive nature of pain perception (Mancini et al. 2022; Mulders et al. 2023), revealing a diffuse neural architecture associated with anticipatory, threat-based, precision-weighting (Eckert et al. 2022; Chen and Wang 2023). Yet, in their current form, these Bayesian models remain largely confined to stimulus-response paradigms, reflecting single-scale experimental contingencies. Crucial to the adequate translation of Bayesian accounts of the multiscale phenomenon of pain is the extension in scope of the Bayesian approach to other levels of ontology (e.g. behavioural and psychological). Theory-driven computational models in pain research have explored these realms (Tabor and Burr 2019; Kiverstein et al. 2022), yet the realisation of such multiscale models in practical terms—incorporating subsystem functioning (e.g. immune, endocrine and neural systems) as well as supersystem dynamics (e.g. social networks and goal motivation)—requires adequate framing. Our proposed framework may function as such a framing that could help researchers and clinicians to identify the scale at which their inquiry is located and track the scales at which their intervention acts.

## A unified approach

We submit that taken together, anthropological inquiry alongside the multiscale ontology and the multilevel epistemology of Bayesian computational psychiatry provides a unified approach to the investigation and intervention in pain. This method combines the anthropological stance described above, with the computational framework outlined in the previous section. Here, we focus our application on a clinical case described in the ethnographic work of Kleinman (Good et al. 1994) (Box 1). Under the lance of the proposed method, we indicate how such phenomenological observations may be translated into both multiscale and multilevel investigation and intervention for persistent pain. We do this by showing how, in taking the anthropological stance, one can leverage the ontological and epistemology of computational psychiatry to guide one’s action.

(Kleinman 1994) is meticulous in his existential accounts of individual experiences of pain, articulating the microcosmic constituents of a local world in pain (Box 1), while setting these lifeworlds in a sociocultural frame. Yet, as noted by Kleinman himself, the potential limitation of traversing between the microcosm and the macrocosm in this way, is a ‘transmogrification’ of the experience. At risk, is a sacrifice of the nuance of correspondence, the texture of pain, in pursuit of an explanation that extends and dehumanises the sufferer. Our task then, is to preserve the rich texture of local lifeworlds in translation, to affirm the humanity to which experience belongs.

The case study (Box 1) provides excerpts from the experiences of a 31-year-old Ph.D. biochemistry researcher who has suffered severe pain for 4 years following a car accident. In applying an anthropological stance, underwritten by the principles of computational psychiatry, the aim is not to recreate an abstract, computational model of the lifeworld. It is to provide an apparatus with which to take coherent action.

Box 1. **Phenomenological excerpts of a clinical case: Case 1 (Kleinman 1994: 175–182).**

‘*At the hospital they diagnosed a concussion and I had broken a few small bones in my foot…Otherwise, there was nothing else injured. But right away I could feel the pain…And that started the whole process. Four years of pain, surgeries, casts, more pain, more tests, more drugs, more surgeries, bad surgical effects, and now this constant pain…And me, us-our lives ruined. All for what?*

‘Pain patients like me are a sign of the failure of the medical care system, of something terribly wrong at the core’.

‘My generation of researchers has moved on…I have to prove that I can put in a full research day, complete projects, that I am like everyone else’.

‘It’s distressing to be viewed as a risk. I used to be seen as a rising star…There is a constant stress of producing, no matter how I feel, to be productive, act successful, present myself as healthy. But I am not healthy…Have to pretend’.

‘Now, they [her family] get pretty angry at me. They simply don’t understand what is going on. My sickness has really affected them’.

### An apparatus for translation

**Figure 1.**
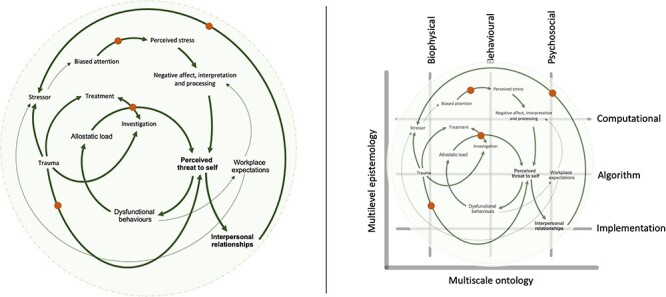
A principled method for investigation and intervention in pain based on an anthropological stance and the ontology and epistemology of computational psychiatry. Left panel. A lifeworld in pain: a schematic representation of ‘how’ the lines of a lifeworld may become entangled, unveiling relevant points of correspondence (bold), which facilitate directed inquiry (orange dots) into ‘why’ the experience of pain occurs in this way, appealing to levels of explanation. Right panel. A computational framing of a lifeworld: the *X* axis represents the three ontological levels of organisation that are modelled in Bayesian modelling psychiatry. The *Y* axis represents Marr’s three epistemological levels of explanation that Bayesian modelling psychiatry refers to explain their modelling rationales. Pain, understood as part of an inter-subjective lifeworld, incorporates processes that are located across the scales of ontology (X) and levels of epistemology (Y).

Identifying the processes that make up an individual’s lifeworld, from which pain emerges becomes the primary focus of inquiry: attending to the lines and the nature of their correspondence. Promoting person-centred investigation and intervention, this principled method should be oriented around the scales of influence and the levels of explanation (Fig. 1, right panel). Specifically, the computational framework outlined provides an adumbrate for (i) the ways in which lines of the lifeworld cross scales of influence, and the strength of their correspondence (scale) and (ii) what kind of explanation one ought to adopt to make sense of the particular lifeworld and to guide decision-making (level). Here, we observe the lifeworlds of anthropology as organised around the ontology and epistemology of computational psychiatry (Fig. 1).

In the case study outlined (Box 1), the phenomenological references relay a tangle of lines that provide partial insight into the individual’s lifeworld. Evolving over time, the lines, denoting the processes through which pain emerges, permeate ontological scales, unbounded (Fig. 1, left panel). Attending to the lines and the way they change their affiliation resembles an ongoing education (Ingold 2018). As researchers or clinicians, we aim to learn the contingencies of experience, establishing ‘what is at stake’ for the individual.

For the 31-year-old woman in Case 1, the experience of pain does not begin with broken bones in the foot, but rather reaches out to an embodied history (past trauma, psychological traits, genetic and phenotypic dispositions) and an anticipated future (professional aspirations, interpersonal commitments and personal identity). The impact of the acute trauma (broken bones) is to draw specific lines into correspondence, informing the perception of threat to the self in the present. Over time, moment-to-moment shifts in correspondence reflect changing affiliations between processes as the individual navigates a dynamic landscape, embodied and embedded within it. This may involve a persistent stress response triggered by on-going somatosensory information, workplace expectations (‘It’s distressing to be viewed as a risk. I used to be seen as a rising star…’) and interpersonal relations (‘Now, they [her family] get pretty angry at me. They simply don’t understand what is going on’). It may also involve dysfunctional behaviours (‘There is a constant stress of producing, no matter how I feel, to be productive, act successful’), which in turn has a bearing on the perception of threat. Similarly, when investigations and interventions remain repetitive and seemingly stagnant (e.g. ‘…pain, surgeries, casts, more pain, more tests, more drugs, more surgeries, bad surgical effects, and now this constant pain…’), the correspondence between lines constricts, reflecting a narrowing of attention, precisely attuned to an ongoing perception of threat to the self. At risk, within these constricted affiliations, is a loss of coherence between the local lifeworld of the individual and her wider environment (e.g. ‘[I] present myself as healthy. But I am not healthy…I [h]ave to pretend’).

Given this complex system of correspondence, a dynamic approach to investigation is required; untethered to scales of influence (Fig. 1, right panel). Importantly, this does not necessitate radical new approaches to intervention. Instead, it demands agile attention trained on the lines of a lifeworld that evokes an iterative approach to multifaceted intervention. Much like the melodies of a jazz ensemble, interventions are seen as processes that become part of a lifeworld, attempting to reconfigure the affiliations of lines, and in so doing altering the experience of pain. Here, irrespective of the precipitating event, whether traumatic or idiopathic, the individual experiencing pain is considered at the level of the lifeworld, in which tissue integrity and anti-inflammatory medication present just one possible affiliation between processes that never occur in isolation. Expanding the scope of attention to consider multiscalar affiliations between the potential lines of influence (e.g. historic trauma, interpersonal relationships and stressor exposure) promotes interventions (psychological and behavioural therapies, medication, pain-relevant education, child support and workplace adaptations) that are deployed in an integrated, specific, and timely manner; constantly attuning to the shape of an individual’s lifeworld.

## Conclusion

We have proposed that a coherent approach to the investigation and intervention of the experience of pain, whether acute or chronic, requires an anthropological stance, supported by the Bayesian apparatus of computational psychiatry. In outlining lifeworlds in pain, focussing on the dynamic correspondence that defines the shape of pain over time, and appealing to ontological and epistemological frames of reference, we are able, as patients, clinicians and researchers, to attune to the experience of pain. In so doing, we establish the principles for effective knowledge translation (a multi-scale ‘going along-with’), recognising that, as Kleinman (1997) puts it, ‘[e]xperience is emergent, not pre-formed. It changes. It goes on and on…’ and we must go along with it.

## Data Availability

No new data were generated or analysed in support of this research.
